# The Effects of the COVID-19 Pandemic on Teleworking and Education in a Romanian Higher Education Institution: An Internal Stakeholders Perspective

**DOI:** 10.3390/ijerph18158180

**Published:** 2021-08-02

**Authors:** Ştefan-Alexandru Catană, Sorin-George Toma, Andreea Barbu

**Affiliations:** 1 Faculty of Business and Administration, University of Bucharest, 030018 Bucharest, Romania; tomagsorin62@yahoo.com; 2 Faculty of Entrepreneurship, Business Engineering and Management, University Politehnica of Bucharest, 060042 Bucharest, Romania; barbu.andreeab@yahoo.com

**Keywords:** teleworking, education, higher education institution, internal stakeholders, well-being, physical inactivity, household activities, Faculty of Business and Administration

## Abstract

The COVID-19 pandemic has created the conditions for the expansion of teleworking (TW) in numerous sectors and organizations, and higher education institutions (HEIs) have had to adapt to this context. This paper aims to identify and analyze five factors (technology, individual involvement and skills, physical inactivity, psychological well-being, and household activities) that influence the effort and results in TW and education (E) in HEIs from the perspective of their key internal stakeholders. The data were gathered by a mix of qualitative and quantitative research methods, such as interviews and surveys. They were analyzed and interpreted through factorial analysis that uses the presentation of the main components as an extraction method, with the Varimax rotation method adopting Kaiser normalization, and processed with SPSS statistical software. This study shows that the effort and results of the key internal stakeholders of HEIs are influenced by the five factors. In this respect, students’ results are negatively influenced by technology and physical inactivity factors. Moreover, the efforts of auxiliary and non-teaching staff are highly positively influenced by the psychological well-being factor and their results are positively influenced by the individual involvement and skills factor and negatively influenced by the household activities factor.

## 1. Introduction

Last year brought exceptional changes and unprecedented challenges not only to the global economy, but also to human civilization. March 2020 will remain a turning point in the history of humanity as numerous and severe nationwide lockdowns have entered into force around the world since the emergence of the COVID-19 pandemic. A huge number of organizations worldwide (e.g., multinational and transnational corporations, small and medium enterprises, public institutions) have started to send their employees home, and therefore created the conditions for the expansion of a widespread phenomenon called TW, known also as work-for-home, remote work, home-office, home-based work, telecommuting, or smart-working [1,2,3].

The increasing use of digitalization and the diffusion of the disruptive and rapid advances in information and communication technologies have highly facilitated the implementation of TW in numerous sectors, such as public administration, insurance, banking, or higher education, and contributed to the flexibilization of the labor market [4,5,6]. In essence, they have led to the birth of new types of organizations and working methods and revolutionized the deployment of the working processes (e.g., virtual teams). Information and communication technologies enable work tasks to be accomplished not only more quickly and consistently with lower efforts, but also at distance. TW consequently involves work undertaken using information and communication technologies and carried on outside the specific workplace. It is worth emphasizing that TW has mostly been the advantage of well-paid employees [7] and is traditionally found in high-skilled, white-collar jobs [8].

The idea of TW was introduced by Jack Nilles (1975) to name a relatively new mode of alternative work arrangements and it widely spread primarily at the beginning of the 21st century as a new form of labor organization that could provide a solution to many individual, social, and organizational problems [9,10,11]. Although there is no internationally recognized definition, telework is considered as using “information and communication technologies, such as smartphones, tablets, laptops and/or desktop computers, for work that is performed outside the employer’s premises” [4] (p. 1). In essence, telework has two main characteristics: involves performing a professional activity remotely—from home or another location—using information and communication technologies [8] and is restricted to employees only [12]. The focused literature analyzed TW from a wide variety of viewpoints (e.g., individual, organization, society), revealing both its advantages and disadvantages [11]. For decades, working from home, a form of TW, has been promoted as one of the management policies that bring benefits such as cost savings [13], work flexibility, time-planning skills [14], reduced employee’s turnover [15], less absenteeism [15], reductions in office space requirements [16], better work–life balance, higher work autonomy and morale [17], retrieve of temporal and spatial constraints in daily activities schedules [18,19], the potential to harmonize the different facets of people’s lives, permitting space and time management [20,21], improved productivity, and reduced informal communication [22]. For example, work flexibility allows teleworkers to address various personal and/or family needs, such as healthcare, eldercare, or childcare. However, other authors reveal the negative impact of TW as follows: overloading with work [11,23,24], connectivity problems [25], physical inactivity [26], psychological stress from technology dependency [27,28,29], lack of supervisors’ physical control over the employees [30], possible lack of trust between managers and their subordinates [30], low level of interaction between employees and their colleagues [31], work-related problems invading personal life [32,33], social isolation [34], various health complications (e.g., cardiovascular disease, cholesterol increase) [35], and the techno-insecurity of data [29,36].

As the COVID-19 pandemic has dramatically affected and is still affecting the global higher education system, TW has become one of the most popular responses to this outbreak as educational services and scientific research can be reasonably performed at home or online [37,38,39]. In this respect, more than 40% of teachers from the European Union used telework in 2018, one of the highest prevalence within the knowledge and information and communication technologies-intensive sectors [40]. However, educational services remain open to both home-based work and face-to-face activities as work flexibility increases engagement and job satisfaction, and improves the well-being of the workers [41,42,43]. As a broad and multifaceted concept, well-being is defined as “the balance point between an individual’s resource pool and the challenges faced” [44] (p. 230). It is linked with similar other concepts (e.g., happiness, contentment, wellness), expresses the positive feelings experienced by someone, and encompasses several types such as psychological or social well-being [45,46]. For example, there is a positive and significant relationship between psychological well-being and self-esteem [47].

Education is defined as the act or process of imparting or acquiring general knowledge, developing the powers of reasoning and judgment, and generally of preparing oneself or others intellectually for mature life [48]. For individuals, education may provide employment, earnings, health, and poverty reduction [49]. Education is also linked to the improvement of public health, allows for nourishing psychosocial environments that support human development (e.g., sense of control and social support), work (e.g., working conditions and income), and helps to foster health knowledge and behaviors [50,51].

HEI is a term used in Europe to designate organizations providing higher, postsecondary, tertiary, and/or third-level E [52]. A university represents both a higher education learning, teaching, and researching institution and a community of stakeholders (e.g., teachers, researchers, students, auxiliary and non-teaching staff). The relationships with its stakeholders highly influence the success of a HEI. Stakeholders are defined as all those organizations, networks, and private people that are able to influence the objectives of a given organization [53,54]. For a HEI, stakeholders include a plethora of elements participating and/or benefiting from the provision of educational services such as teachers, students, parents, companies, or society. HEIs classify stakeholders as either internal or external [55]. Internal stakeholders are the rector, the deans, teachers, students, faculty representatives, as well as auxiliary and non-teaching staff, whereas external stakeholders comprise partners and customers [56].

In HEIs, e-learning has turned into an alternative to traditional face-to-face education systems for those persons that want to study, but have to go through a distance to university, or need more flexibility for different reasons [57]. Moreover, studies have shown that TW in academia has been developed over the last two decades and it does not exclude face-to-face activities that could be combined with the traditional way of teaching thus forming blended learning [58]. In Romania, the crisis generated by the COVID-19 pandemic made TW not only an option, but also a necessity for continuing the educational process in HEIs [59]. As a result, online education delivery continues to develop rapidly and expand widely, gaining support across all educational sectors [60,61].

Recent researches have investigated how internal stakeholders in the higher education sector are affected by TW. In a comparison study between academic teleworkers and non-teleworkers, Tustin [62] found that academics appreciate TW and their students are more satisfied with academic support from telecommuters than non-telecommuters. Other studies considered that teleworkers felt social and psychological well-being [17]. They are more productive, more satisfied with their work, and less stressed compared with work at the office [63]. Although non-teaching staff appreciated the life satisfaction and the possibility of managing a family and doing their home stuff alongside their work, they reported that it is difficult to set properly the work and leisure time [64].

The above considerations show that there is a close relationship between internal stakeholders, TW, and E in HEIs. Firstly, the studies carried on in this field revealed a high level of satisfaction from teachers, students, and auxiliary and non-teaching staff towards TW and online E [52,53,54], thus being mutually beneficial to the stakeholders. Secondly, these concepts focus on the economic, technological, social, and human dimensions, and TW is specific to employees. Thirdly, both internal stakeholders and TW activities contribute to the development of HEIs [65].

Based on the theoretical framework previously displayed, two principal research objectives were set up:

**Objective** **1** **(O1).** *To identify and analyze some of the main factors that influence TW and E in HEIs*.

**Objective** **2** **(O2).** *To present the perspective of the key internal stakeholders of HEIs on these factors*.

**Figure 1:**
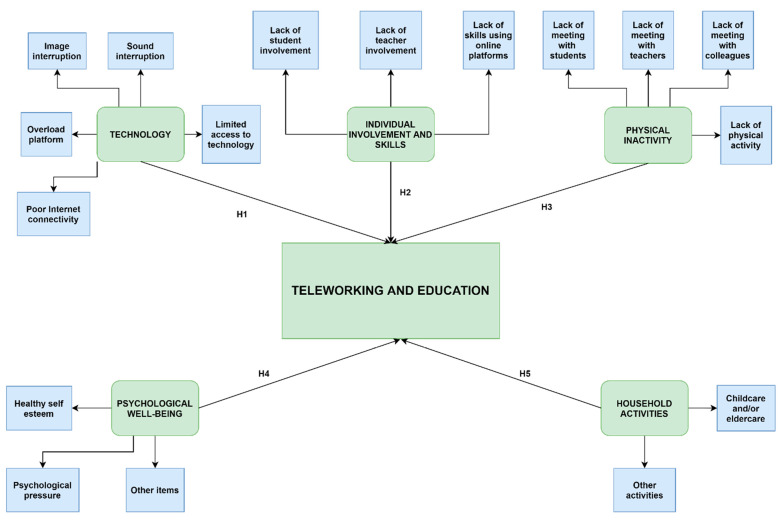
Research model.

The authors have designed and empirically tested a theoretical model to explain the influence of five factors (technology, individual involvement and skills, physical inactivity, psychological well-being, and household activities) on TW and E in HEI (Figure 1). Each factor is defined through a different number of items. As processes, TW and E involve the existence of inputs (effort) and outputs (results). Therefore, the dependent variables are the effort and results of the key internal stakeholders (students, teachers, and auxiliary and non-teaching staff) during TW and E in HEIs and the independent variables are the five factors previously mentioned. This study addresses the context in which TW is related to teachers and auxiliary and non-teaching staff, while E is related to students and teachers.

This research attempts to measure the influence, either positive or negative, of the five independent variables on the effort and results of the key internal stakeholders during their activities in the academic environment. In this respect, the authors used statistical tools such as the Varimax rotation method with Kaiser normalization, Cronbach’s Alpha coefficient, t-test, Levene’s Test, and Pearson coefficients.

Starting from the above objectives, the following six research hypotheses were formulated:

**Hypothesis** **1** **(H1).** *Technology factor negatively influences the internal stakeholders’ results in TW and E in HEIs*.

**Hypothesis** **2** **(H2).** *Individual involvement and skills factor positively influences the internal stakeholders’ results in TW and E in HEIs*.

**Hypothesis** **3** **(H3).** *Physical inactivity factor negatively influences the internal stakeholders’ results in TW and E in HEIs*.

**Hypothesis** **4** **(H4).** *Psychological well-being factor positively influences the internal stakeholders’ effort in TW and E in HEIs*.

**Hypothesis** **5** **(H5).** *Household activities factor negatively influences the auxiliary and non-teaching staff’s results in TW in HEIs*.

**Hypothesis** **6** **(H6).** *The key internal stakeholders consider that the traditional educational system (face-to-face) represents the best way to carry out the educational process (this hypothesis does not appear in Figure 1)*.

Against this background, the paper aims to identify and analyze the above five factors that influence the effort and results in TW and E in HEIs from the perspective of their key internal stakeholders. To accomplish these purposes, the authors used a mix of qualitative and quantitative research methods (e.g., interviews, surveys) within a Romanian HEI.

This study is conducive to the development of the literature on TW and E in HEIs. It pinpoints and interprets some of the main factors that affect TW and E from the key internal stakeholders’ point of view.

The paper is structured as follows. Section 2 presents materials and methods. Results and a discussion are presented in Section 3 and Section 4, respectively. Section 5 illustrates the conclusions, along with their limitations and research perspectives.

## 2. Materials and Methods

Firstly, the authors looked for information through desk research. Several secondary data (e.g., books, articles) from the fields of economics and business administration were identified and collected from electronic databases (e.g., Springer) and libraries (e.g., the Central University Library Carol I of Bucharest). Secondly, these data were classified, analyzed, and synthesized. Thirdly, the authors chose the populations to be addressed starting from the fact that the University of Bucharest has decided to carry out the educational process mostly online, since March 2020. They selected the undergraduate program of the Marketing specialization within the Faculty of Business and Administration, University of Bucharest, due to the following reasons:

- Starting with March 2020, a high proportion of the educational process has been provided through online platforms.
- The number and the size of the internal stakeholders (Table 1, Table 2 and Table 3) allowed the deployment of both comprehensive exploratory and descriptive research, and the use of both qualitative and quantitative research methods. Based on the literature review, three specific groups (students, teachers, and auxiliary and non-teaching staff) were identified as the key internal stakeholders. The respondents were males and females as no one declare being non-binary.
- Two out of three authors are teaching various disciplines to students from all three years of study composing this undergraduate program.

Fourthly, in order to accomplish the objectives and test the hypotheses of the paper, the authors used mixed methods research [66,67], namely qualitative (e.g., in-depth interviews) and quantitative (e.g., surveys). The fieldwork research was conducted between 10 and 24 of February and 1 and 28 of March 2021. In the first period, 12 in-depth interviews, both face-to-face and phone interviews, were carried on with people representing the three groups to identify the main themes of the questionnaires. Interviews were semi-structured, lasted around 30 min, and covered a plethora of factors that influence TW and E as follows: individual factors, job factors, organizational factors, family/home factors [68], and environmental, legal, and safety factors [69].

The six research hypotheses were tested through an online questionnaire applied to three different populations: 349 students, 39 teachers, and 9 auxiliary and non-teaching staff. The relatively long time allowed the deployment of the research on the whole populations, given that the survey participation was voluntary. After receiving, centralizing, and systematizing the data gathered online, 334 questionnaires were validated from students (15 out of 349 sent incomplete responses or did not respond), 35 questionnaires from teachers (2 out of 39 were on child care leave and did not telework and 2 out of 39 sent incomplete responses or did not respond), and 7 questionnaires from the auxiliary and non-teaching staff (2 out of 9 were on child care leave and did not telework). The response rate was: 95.7% in the case of students, 89.7% in the case of teachers, and 77.7% in the case of auxiliary and non-teaching staff, which are higher than the norm of 56% for researches utilizing a questionnaire survey [70]. In the case of students, most respondents were female (59.98%) with an average age of 21.55 years, close to the gender structure of the program (Table 1). In the case of teachers, most respondents were female (54.29%), mostly aged between 31 and 40 years old (34.3%) and between 41 and 50 years old (31.4%), close to the gender and age structure of the program (Table 2). Most respondents were associate professors (28.6%) and assistant professors (22.9%) and had at least 5 years of work experience within the faculty. In the case of auxiliary and non-teaching staff, most respondents were female (77.78%), mostly aged between 31 and 40 years old (42.86%), and had an average of 12.28 years of work experience within the faculty. In their final form, the questionnaires comprised 28 items in the case of students, 27 items in the case of teachers, and 24 items in the case of auxiliary and non-teaching staff, measuring five factors as follows: technology, individual involvement and skills, physical inactivity, psychological well-being, and household activities. The questionnaires also included socio-demographic items (gender, age, work status, residence, income, marital status). The multi-item factors were measured on a five-point Likert scale where 1 = strongly disagree and 5 = strongly agree.

Fifthly, the collected data were interpreted through the factorial analysis that uses as an extraction method the presentation of the main components, along with the Varimax rotation method using Kaiser normalization [71,72], and processed with SPSS statistical software (Version 23, IBM, New York, NY, USA).

## 3. Results

To identify the factors that influence TW and E in HEIs from the students’ perspective, an analysis of the items was performed. The analysis revealed the existence of four main types of factors: technology, individual involvement and skills, physical inactivity, and psychological well-being (Table 4). The values of the Cronbach’s Alpha coefficient were determined to measure the internal validity of the questionnaire, and exceeded the threshold of 0.7, which shows a good internal consistency of the tested items [73].

After investigating the teachers’ group, the same four factors were identified (Table 5). Testing the internal consistency of the items revealed a low score of 0.564 for the factor related to the adaptation to the operation of online platforms, which was excluded from the following analysis. Although the factors related to involvement and physical inactivity obtained values of Cronbach’s Alpha coefficients between 0.6 and 0.7, there are studies that state that a factor above 0.6 still reflects an acceptable consistency of these items considered [74].

In the case of the group formed by the auxiliary and non-teaching staff of the faculty, three factors were identified: technology, physical inactivity, and household activities (Table 6). Testing the internal consistency of the items revealed a score of over 0.7 in the case of Cronbach’s Alpha coefficients, which shows a good consistency of the items considered [73].

As can be seen from the Table 4, Table 5 and Table 6, the technology factor includes, on the one hand, the technical aspects regarding the internet connection and functionality of the platforms, and on the other hand, the skills of using the software. In addition, the authors considered necessary to separately analyze the devices and online teaching platforms used by internal stakeholders. In this respect, all students used their own electronic devices to engage in the online educational process. Most of them possess a smartphone and a laptop (46.71%), and some of them (23.65%) have only a laptop. Moreover, most teachers have electronic devices from their own sources, with the faculty allocating only six laptops for them. In the case of the auxiliary and non-teaching staff, all of them handle laptops, the faculty providing laptops for six of them. Additionally, by analyzing the most used online teaching platforms, the results demonstrate that each group predominantly utilizes four platforms: Google Meetings, Zoom, Moodle, and Microsoft Teams (Table 7).

However, certain technical problems have occurred for all the three investigated groups during the use of the above online teaching platforms. They pointed out that they have often had problems with the overload of the platforms. This item affected to a large extent the quality of the educational online process (50.6% of students and 20% of teachers). Poor internet connectivity is another item that negatively influenced the quality of the educational process. The problems generated by it led to the interruption of sound and loss of information during the educational process (34.7% of students and 11.4% of teachers). On the other hand, image interruption did not alter the quality of the online educational process.

The influences of technology and psychological well-being factors are perceived differently by students depending on the gender of respondents or whether or not they are employed during this period (Table 8). More than that, the individual involvement and skills factor is perceived differently by students depending on their gender. The respondents consider that these three factors influence more the quality of the online educational process. Moreover, students perceive differently the level of effort made during this period depending on their gender.

The students from the first two years of study, who are not employed, are more sensitive to how technology and psychological well-being factors influence the online educational process. The results show a weak negative correlation between these variables and the specified factors (Table 9). To interpret the correlations, the values of the Pearson coefficients were analyzed, using the guide developed by Evans (1996): very weak correlations have values below 0.2, weak correlations have values between 0.2 and 0.4, moderate correlations are characterized by values between 0.4 and 0.6, strong correlations have values between 0.6 and 0.8, while correlations with values above 0.8 are very strong [75].

In the case of teachers, the results demonstrate that their age negatively influences the factors related to the involvement in the educational process (R = −0.371, *p* < 0.05). The youngest considered lack of involvement as a general problem that affects the whole educational process in the online educational process (Table 10). The items related to physical inactivity affect teachers’ psychological well-being as there is a weak positive correlation (R = 0.346, *p* < 0.05). Physical inactivity also influences other items related to psychological well-being such as the adaptability level to the online educational process. In this respect, a lack of physical inactivity led to a lower adaptability level (R = 0.418, *p* < 0.05).

In addition, through independent samples t-tests, the authors checked whether the gender of teachers influences the analyzed variables. The results of these tests show that age is not responsible for how these variables change (Table 11).

In the case of auxiliary and non-teaching staff, the outcomes show that their age or seniority in the institution does not influence the effort or results obtained (Table 12). In contrast, the results obtained in the online educational process are strongly influenced the household activities (R = 0.917, *p* < 0.01), while the psychological well-being factor strongly influences the effort made by auxiliary and non-teaching staff in TW and E processes (R = 0.801, *p* < 0.05).

In addition to these findings, students (41%) and auxiliary and non-teaching staff (57.1%) consider that the best way to carry out the educational process remains the traditional (face-to-face) system. Most teachers (54.29%) consider that the best way to carry out the educational process is blended learning (Table 13).

## 4. Discussion

All of the faculties from the University of Bucharest pivoted to online classes and temporarily jettisoned in-person classes, from March 2020 until the present (June 2021). They did so as a way of embracing and implementing social distancing as one of the main interventions recommended within the COVID-19 pandemic. In fact, many HEIs during this critical period resorted to online instruction as a solace to ensure and salvage their teaching, learning, and research continuity, and as means to comply with social distancing [64]. As highlighted in the theoretical framework section, the practice of deploying social distancing measures, such as closing learning institutions like schools during pandemic outbreaks, seems to be common. Scholars have produced a short review of university closures due to COVID-19 and point out that, due to the COVID-19 pandemic, many universities across the globe have canceled or postponed their academic activities, and have, consequently, transitioned to online educational platforms [76].

Based on the factor analysis, the results of our research illustrated some of the advantages and disadvantages of TW and online E, during the COVID-19 pandemic. The authors identified and analyzed five factors that affect TW and E in a HEI: technology, individual involvement and skills, physical inactivity, psychological well-being, and household activities. Accordingly, while previous studies described many items related to experiences of TW and E, generally [77], our study customized these results in the context of the COVID-19 pandemic.

This study highlighted that the technology factor negatively influences the internal stakeholders’ results in TW and E in HEIs. In the case of students, the obtained results in the online educational process are rather modest, being negatively influenced by the technology factor (R = −0.179, *p* < 0.01). The technology factor does not influence both the teachers’ results in TW and E and the auxiliary and teaching staff’s results in TW in HEIs. These results are in line with previous researches that discuss technology as a determinant of TW [78], including the educational services [79]. Some of them underline the advantages of the technology factor, such as flexibility of working hours and the possibility to work during the most productive time [80], whereas others emphasize its disadvantages such as lower productivity when people are using poor information and communication technologies infrastructure [81], lack of skills to deal with increasingly sophisticated technology [82], and possible loss of data security [16], which are also found in HEIs. This research shows that individual involvement and skills factor positively influences the internal stakeholders’ results in TW and E in HEIs. In the case of auxiliary and non-teaching staff, their results are positively influenced by this factor (R = 0.228, *p* < 0.01). On the other hand, the lack of involvement of both teachers and students led to an absence of interactivity between these two groups, which puts psychological pressure on both sides. Other studies highlight that, during the online educational process, teachers reported a sense of worry and concern for students and deeply felt their absenteeism [83]. Moreover, in other researches, teachers stated that the COVID-19 pandemic increased student anxiety and parental stress [84].

The results confirmed the third hypothesis that physical inactivity has a negative influence on the internal stakeholders’ results in TW and E in HEIs due to the lack of physical meetings between them. In the case of students, the outcomes obtained in the online educational process are rather modest due to the physical inactivity (R = −0.166, *p* < 0.01), in line with other studies [85]. With the outbreak of COVID-19, social distance together with its coeval, physical distancing, has emerged not only as a mantra but also as a prism through which coronavirus is viewed [86]. With social distancing and quarantine strategies, people spend more time at home, with less opportunity for an active lifestyle [87], leading to the appearance of health threats, such as occupational and cardiovascular diseases [82], which are also found in HEIs. In terms of the influence of the psychological well-being factor on the internal stakeholders’ effort in TW and E in HEIs, the fourth hypothesis was validated. In the case of the auxiliary and non-teaching staff, their effort is positively highly influenced by this factor (R = 0.801, *p* < 0.01). Psychological well-being associated with TW has been studied in a different context [88], including HEIs [89,90]. Other studies reveal that, during the COVID-19 pandemic, teleworkers’ stress was caused by new factors such as health and life threats, numerous restrictions, and recommendations due to the epidemic state (stay-at-home, closure of many institutions), isolation, lack of social support [91], inability to connect effectively with employing organization [92] and a reduced sense of belonging to the organization [15,82]. Satisfaction with life and the affective component of psychological well-being tend to correlate because both are influenced by the assessment made by people about activities and circumstances in which life is carried out [93]. In accordance with other studies, the persons who attained a tertiary level of education, such as teachers and students, experience relatively more negative consequences from TW and E on relationships with colleagues [31].

The results confirmed the fifth hypothesis that the household activities factor negatively influences the auxiliary and non-teaching staff’s results in TW in HEIs. In this respect, carrying out work tasks at their own homes, at the same time as carrying out household chores, creates a great psychological pressure among the auxiliary and non-teaching staff, who now perceive a much greater effort than in the period in which they worked at their workplace. The fact that they are tempted to deal in parallel with household chores, not only what they have to do for professional activity, eventually leads to record poorer results from a professional point of view, as their concentration is no longer 100% directed to what they have to do in this regard (R = −0.766, *p* < 0.05). Previous studies in this field of research indicated that women pay more attention to family duties. Thus, they are attracted more to home-based telework, which assists them in balancing work and family responsibilities [94]. One of the disadvantages of TW in HEIs to the household activities is the fact that women find themselves multitasking due to their multiple domestic responsibilities [95].

The study invalidates the sixth hypothesis. The traditional system (face-to-face) is considered the best way to carry out the educational process by students (41%) and auxiliary and non-teaching staff (57.1%), whereas teachers (45.71%) believe that blended learning (mixed system) is the best way, in accordance with other researches [96]. In addition, the study shows that the three key internal stakeholders used mainly laptops or smartphones as their own electronic devices for carrying out online activities. The most used online platforms during the COVID-19 pandemic were Google Meetings and Zoom. Moreover, previous studies revealed that the demand for video conferencing apps has surged during the TW [97] and the most used online educational platforms were Microsoft Teams and Zoom [98].

This study leads to several practical implications. Firstly, the government may support and promote a culture of TW and online E through investments in modern technologies. Secondly, HEIs may sustain their key internal stakeholders by delivering them electronic devices, ensuring up-to-date online educational platforms, and organizing training courses for developing technological skills and psychological counseling. Thirdly, HEIs may involve other stakeholders through partnerships (e.g., companies) to improve the quality of the online educational process.

Concerning future lines of research, it might be relevant to expand the study on other internal stakeholders and external stakeholders, such as alumni, statutory authorities, local and national government bodies, local and regional communities, local businesses, committees, and elected officials. Since this study has been based on some of the factors that influence the internal stakeholder’s effort and results in TW and E in HEIs, future researches should be conducted to identify and analyze other important factors. Moreover, they might take into consideration the possible correlations among the items related to these factors. Other researchers may monitor the extent to which these and other factors are valid outside of this pandemic context. Another limitation of our study is the size and the structure of the populations, as these are representative only for the Marketing specialization within the Faculty of Business and Administration from the University of Bucharest. A larger and more representative population should be analyzed for future researches.

## 5. Conclusions

The appearance and expansion of the COVID-19 pandemic have dramatically changed the way activities are carried out in organizations from various sectors. From a theoretical point of view, this paper contributes to the enrichment of the literature on TW and E in HEIs. It provides a theoretical model that brings some clarifications regarding the perspective of their key internal stakeholders on TW and E. In addition, the paper presents the connection between these concepts in HEIs, highlighting the fact that they focus mainly on the same dimensions: economic, technological, social, and human. It also shows that TW and E lead to the development of HEIs through the active involvement and participation of their key internal stakeholders (students, teachers, and auxiliary and non-teaching staff).

From a practical point of view, TW and online E in a HEI should be implemented by taking into account the needs and expectations of its key internal stakeholders. This paper identifies and investigates some of the factors (technological, individual involvement and skills, physical inactivity, psychological well-being, and household activities) that influence the internal stakeholder’s effort and results in TW and E in HEIs, by taking into account their opinions. Firstly, this study shows that the students’ results are negatively influenced by the technology factor and physical inactivity. Secondly, it demonstrates that the efforts of auxiliary and non-teaching staff are highly positively influenced by the psychological well-being factor and their results are positively influenced by the individual involvement and skills factor and negatively influenced by the household activities factor. Thirdly, students and auxiliary and non-teaching staff consider that the traditional system (face-to-face) represents the best way to carry out the educational process, while teachers state that blended learning (mixed system) is the best way.

Last but not least, there is a need for future studies related to the factors that influence TW and E in HEIs. This should be sustained by the technological advances, on one hand, and, on the other hand, by the psycho-demographic changes.

## Figures and Tables

**Table 1:** Year of study, gender, and number of students within the Marketing specialization, undergraduate program.

Year of Study	Number of Students	Gender	
		Male	Female
I	128 (36.67%)	51	77
II	124 (35.53%)	57	67
III	97 (27.80%)	43	54
Total	349 (100%)	151 (43.26%)	198 (56.74%)

**Table 2:** Gender, age, title, and number of teachers within the Marketing specialization, undergraduate program.

Title	Number of Teachers	Gender		Age				
		Male	Female	21–30	31–40	41–50	51–60	61–70
Professor	5	3	2	0	0	2	2	1
Associate professor	12	8	4	0	2	9	1	0
Lecturer	9	7	2	0	6	1	2	0
Assistant professor	13	1	12	7	5	1	0	0
Total	39 (100%)	19 (48.72%)	20 (51.28%)	7 (17.96%)	13 (33.33%)	13 (33.33%)	5 (12.82%)	1 (2.56%)

**Table 3:** Gender, age, and number of auxiliary and non-teaching staff within the Marketing specialization, undergraduate program.

Number of Auxiliary and Non-Teaching Staff	Gender		Age			
	Male	Female	21–30	31–40	41–50	51–60
9	2	7	1	6	1	1

**Table 4:** Testing data from the students’ group.

Items	Factor Loadings	Factor	EV	% Variance	Cronbach’s Alpha
Sound interruption	0.824	Technology	4.61	35.494	0.809
Image interruption	0.803				
Overload platform	0.746				
Poor Internet connectivity	0.738				
Lack of student involvement	0.853	Individual involvement and skills	1.82	14.045	0.784
Lack of teacher involvement	0.840				
Lack of skills for using online platforms	0.705				
Lack of meeting with teachers	0.917	Physical inactivity	1.54	11.847	0.805
Lack of meeting with colleagues	0.891				
Lack of physical activity	0.590				
Healthy self-esteem	0.799	Psychological well-being	1.05	8.106	0.712
Other items (e.g., motivation, loneliness)	0.787				
Psychological pressure	0.609				

Note: EV—Eigenvalue.

**Table 5:** Testing data from the teachers’ group.

Items	Factor Loadings	Factor	EV	% Variance	Cronbach’s Alpha
Sound interruption	0.918	Technology	3.38	30.797	0.847
Image interruption	0.879				
Overload platform	0.838				
Poor Internet connectivity	0.645				
Lack of student involvement	0.864	Individual involvement and skills	1.36	12.388	0.652
Lack of skills for using online platforms	0.812				
Lack of teacher involvement	0.803				
Lack of physical activity	0.907	Physical inactivity	1.49	74.53	0.657
Lack of meeting with colleagues	0.863				
Lack of meeting with students	0.863				
Psychological pressure	0.878	Psychological well-being	1.88	17.171	0.816
Other items (e.g., motivation, loneliness)	0.650				
Healthy self-esteem	0.602				

Note: EV—Eigenvalue.

**Table 6:** Testing data from the auxiliary and non-teaching staff group.

Items	Factor Loadings	Factor	EV	% Variance	Cronbach’s Alpha
Image interruption	0.974	Technology	2.21	55.37	0.763
Limited access to the technology	0.866				
Overload platform	0.846				
Poor Internet connectivity	0.736				
Lack of meeting with colleagues	0.895	Physical inactivity	4.68	58.57	0.944
Lack of physical activity	0.885				
Care of children or elderly	0.724	Household activities	1.39	17.42	0.877
Other activities (e.g., feeding pets, doing laundry)	0.709				

Note: EV—Eigenvalue.

**Table 7:** Online teaching platforms used by the key internal stakeholders.

Online Teaching Platforms	Students (%)	Teachers (%)	Auxiliary and Non-Teaching Staff (%)
Google Meetings	79.6	60.0	57.1
Zoom	69.8	57.1	55.2
Moodle	64.7	65.7	-
Microsoft Teams	32.4	45.7	12.5

**Table 8:** Independent Samples T-Test for Gender and Employee variables (in students’ case).

		Levene’s Test for Equality of Variances			t-Test for Equality of Means			
		F	Sig.	Equal Variances (Assumed/Not Assumed)	t	df	Sig. (2-Tailed)	Mean Difference
Gender	T	1.380	0.241	assumed	−4.752	332	0.000	−0.47821
	Inv	5.356	0.021	not assumed	−2.667	258.349	0.008	−0.32663
	P In	0.359	0.549	assumed	−1.010	332	0.313	−0.11554
	Pw	5.427	0.020	not assumed	−5.080	263.951	0.000	−0.61597
	Results	3.978	0.047	not assumed	−1.155	308.118	0.249	−0.129
	Effort	2.647	0.105	assumed	−2.827	332	0.005	−0.370
	Comf	1.174	0.279	assumed	2.513	332	0.012	0.291
Employee	T	0.336	0.563	assumed	2.024	332	0.044	0.21025
	Inv	1.965	0.162	assumed	0.387	332	0.699	0.04666
	P In	0.143	0.706	assumed	1.466	332	0.144	0.16829
	Pw	0.293	0.589	assumed	3.875	332	0.000	0.46767
	Results	0.577	0.448	assumed	−0.278	332	0.781	−0.032
	Effort	1.052	0.306	assumed	0.898	332	0.370	0.119
	Comf	1.951	0.163	assumed	−1.428	332	0.154	−0.167

Note: T—Technology factor; Inv—Involvement factor; P In—Physical inactivity; PW—Psychological well-being factor; Results—Level of results appreciation for the online educational process; Effort—Level of effort appreciation for the online educational process; Comf—Comfort level for using electronic devices and platforms; N = 334.

**Table 9:** Correlations between various variables associated with the group of students.

Variables	T	Inv	P In	PW	Adapt	Effort	Results	CW
Age	−0.171 **	−0.035	−0.011	−0.209 **	0.152 **	−0.021	0.087	0.185 **
Year of study	−0.230 **	−0.031	0.006	−0.294 **	0.175 **	−0.038	0.111 *	0.242 **
Work experience	−0.091	0.09	0.024	0.015	0.032	0.132	0.154	0.147
Average income	−0.129 *	0.014	0.064	−0.187 **	−0.029	−0.002	−0.104	−0.073
Family members	0.108 *	−0.034	−0.008	0.009	−0.105	−0.044	−0.067	0.016
T	1	0.331 **	0.255 **	0.399 **	−0.351 **	−0.023	−0.179 **	−0.290 **
Inv	0.331 **	1	0.333 **	0.443 **	−0.201 **	0.014	−0.032	−0.121 *
P In	0.255 **	0.333 **	1	0.454 **	−0.277 **	0.049	−0.166 **	−0.332 **
PW	0.399 **	0.443 **	0.454 **	1	−0.315 **	0.087	−0.065	−0.281 **
CP	−0.203 **	−0.250 **	−0.189 **	−0.286 **	0.419 **	0.170 **	0.168 **	0.415 **
Comf	−0.412 **	−0.229 **	−0.281 **	−0.373 **	0.555 **	0.106	0.276 **	0.455 **
Adapt	−0.351 **	−0.201 **	−0.277 **	−0.315 **	1	0.101	0.249 **	0.410 **
Effort	−0.023	0.014	0.049	0.087	0.101	1	0.086	0.244 **
Results	−0.179 **	−0.032	−0.166 **	−0.065	0.249 **	0.086	1	0.495 **
CW	−0.290 **	−0.121 *	−0.332 **	−0.281 **	0.410 **	0.244 **	0.495 **	1

Note: T—Technology factor; Inv—Involvement factor; P In—Physical inactivity; PW—Psychological well-being factor; CP—Characteristics of the educational process; Comf—Comfort level for using electronic devices and platforms; Adapt—Adaptability level in the online educational process; Effort—Level of effort appreciation for the online educational process; Results—Level of results appreciation for the online educational process; CW—Level of appreciation of the conducting way of the online educational process; N = 334; * Correlation is significant at the 0.05 level (2-tailed); ** Correlation is significant at the 0.01 level (2-tailed).

**Table 10:** Correlations between various variables associated with the group of teachers.

Variables	T	PW	Inv	P In	Effort	Results	CW	Adapt
Age	−0.288	0.095	−0.371 *	0.316	0.092	−0.25	−0.226	−0.059
Seniority	−0.171	0.111	−0.171	0.248	0.249	−0.207	−0.142	0.067
Average income	−0.1	0.161	0.065	−0.005	0.193	0.143	−0.186	−0.069
Family members	0.144	0.287	−0.005	0.069	−0.16	0.176	−0.014	0.119
T	1	0.111	0.205	0.303	0.093	0.096	0.243	0.193
PW	0.111	1	0.066	0.346 *	0.268	−0.073	−0.107	0.219
Inv	0.205	0.066	1	0.088	−0.047	−0.029	−0.088	0.184
P In	0.303	0.346 *	0.088	1	0.008	−0.301	0.023	0.418 *
Effort	0.093	0.268	−0.047	0.008	1	0.17	0.051	−0.023
Results	0.096	−0.073	−0.029	−0.301	0.17	1	0.402 *	0.121
CW	0.243	−0.107	−0.088	0.023	0.051	0.402 *	1	0.343 *
Adapt	0.193	0.219	0.184	0.418 *	−0.023	0.121	0.343 *	1

Note: T—Technology factor; PW—Psychological well-being factor; Inv—Involvement factor; P In—Physical inactivity; Effort—Level of effort appreciation for the online educational process; Results—Level of results appreciation for the online educational process; CW—Level of appreciation of the conducting way of the online educational process; Adapt—Adaptability level in the online educational process; N = 35; * Correlation is significant at the 0.05 level (2-tailed).

**Table 11:** Independent samples t-tests for teachers’ gender.

	Levene’s Test for Equality of Variances			t-Test for Equality of Means			
	F	Sig.	Equal Variances (Assumed/Not Assumed)	t	df	Sig. (2-Tailed)	Mean Difference
T	0.122	0.729	assumed	0.206	33	0.838	0.05757
Inv	0.458	0.503	assumed	0.174	33	0.863	0.06086
Pw	0.123	0.728	assumed	0.327	33	0.746	0.14145
P In	0.299	0.588	assumed	2.018	33	0.052	0.59704
Adapt	0.976	0.330	assumed	0.416	33	0.680	0.207
Effort	9.324	0.004	not assumed	0.700	24.319	0.491	0.197
Results	2.576	0.118	assumed	−0.874	33	0.388	−0.309

**Table 12:** Correlations between various variables associated with the group of auxiliary and non-teaching staff.

Variables	Effort	Results
Age	0.439	0.194
Seniority	0.293	0.387
HA	−0.52	−0.766 *
Inv	−0.279	0.228 *
PW	0.801 *	0.354
P In	−0.348	0
T	−0.421	−0.62

Note: HA—household activities factor; T—Technology factor; PW—Psychological well-being factor; Inv—Involvement factor; P In—Physical inactivity; Effort—Level of effort appreciation for the online educational process; Results—Level of results appreciation for the online educational process; CW—Level of appreciation of the conducting way of the online educational process; N = 7; * Correlation is significant at the 0.05 level (2-tailed).

**Table 13:** The best way to carry out the educational process.

Way of Carrying Out Educational Activities	Students (%)	Teachers (%)	Auxiliary and Non-Teaching Staff (%)
Traditional system (face-to-face)	41	45.71	57.1
Online	19.8	0	28.6
Blended learning (mixed system)	39.2	54.29	14.3
Total	100	100	100

## Data Availability

Data is available on request.
